# 7-Aza-des-*A*-steroids with Antimicrobial and Cytotoxic Activity

**DOI:** 10.3797/scipharm.1303-03

**Published:** 2013-04-14

**Authors:** Melanie Krojer, Marco Keller, Franz Bracher

**Affiliations:** Department of Pharmacy – Center for Drug Research, Ludwig-Maximilians University, Butenandtstr. 5–13, 81377 Munich, Germany.

**Keywords:** Azasteroids, Pyridine, Annulation, Antimicrobial activity, Cytotoxic activity

## Abstract

This paper describes a convenient approach to the 7-aza-des-*A*-steroid (6,6a,7,8,9,9a-hexahydro-5*H*-cyclopenta[*h*]quinoline) scaffold starting from Grundmann’s ketone using two different pyridine annulation protocols. The biological evaluation of the pyridine and pyridinium products revealed that these compounds unexpectedly do not interfere with ergosterol and cholesterol biosynthesis. The pyridinium compound **6** showed significant antimicrobial and cytotoxic activities which are most likely due to its detergent-like structure.

## Introduction

Steroids are of outstanding significance for human metabolism by acting as hormones (androgens, estrogens, gestagens, glucocorticoids, mineralocorticoids) or as an essential part of cell membranes (cholesterol). In fungi and protozoa, ergosterol is an indispensible part of cell membranes, and inhibition of ergosterol biosynthesis is one of the central targets of antifungal and antiprotozoal chemotherapy. Highly active drugs have been designed in the past by introducing new structural motifs into the tetracyclic sterol scaffold by either semisynthesis starting from natural sterols, or by *de novo* synthesis. In glucocorticoids, introduction of halogen substituents (mostly fluorine), additional double bonds, and oxygen-containing groups (alcohols, acetals) resulted in significant enhancement of activity and selectivity [1], whereas the annulation of additional heterocyclic rings (typically at the C-2,C-3 flank) resulted in compounds with various biological 330 M. Krojer, M. Keller, and F. Bracher: activities [2, 3]. Azasteroids containing protonable nitrogen atoms at various positions of the steroid backbone or the side-chain exhibit antifungal activities, with most of them mimicking carbocationic high energy intermediates (HEI) of enzymatic steps in the post-squalene part of ergosterol biosynthesis [4], whereas abiretarone, containing a pyridine ring in place of the aliphatic side chain at C-17, is a potent inhibitor of the enzyme 17α-hydroxylase/C17,20-lyase used therapeutically in cancer therapy due to its androgen-lowering activity [5]. Recently, our group reported about highly cytotoxic 1,4-dioxo-5,10-diazasteroids [1]. 4-Azasteroids containing a lactam moiety in ring A (finasteride, dutasteride) are important inhibitors of the enzyme 5α-reductase in the treatment of benign prostatic hyperplasia and alopecia [6], and lathosterol analogues containing amide groups in the side chain are selective inhibitors of the enzyme lathosterol oxidase in cholesterol biosynthesis [7]. Steroid-derived muscle relaxants like pancuronium bromide contain quaternary piperidinium groups attached to the ring system [8].

**Fig. 1:**
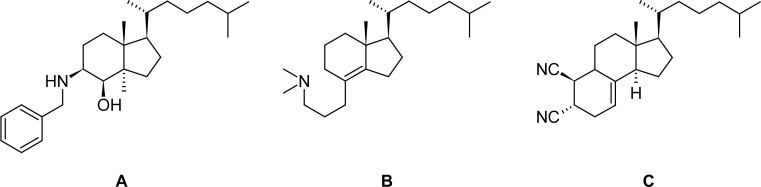
Bioactive compounds derived from Grundmann’s ketone: the aminoalcohol **A**[9] is a selective inhibitor of human sterol Δ8,7-isomerase, the aminopropylindene **B**[10] is an inhibitor of oxidosqualene cyclases from various organisms, the *trans*-dinitrile **C**[11] shows cytotoxicity.

In recent investigations we demonstrated that secosteroids, derived from Grundmann’s ketone (**1**), covering the rings C+D as well as the aliphatic side chain of steroids, and containing amino (**A**) [9] or aminopropyl substituents (**B**) [10], are potent inhibitors of enzymes in sterol biosynthesis, whereas the *trans*-dinitrile **C** shows moderate cytotoxicity, and its *cis*-isomer is inactive [11] (Fig. 1). In the androgen field, a des-*A*-analogue showed separation of androgenic and anabolic activities [12].

Here we report on the synthesis of a novel aza-des-*A*-steroid in which ring B of the steroid backbone is replaced by a pyridine ring. The nitrogen atom is located at a position representing C-7 of steroids, and thus we expected that this azasteroid or its quaternary *N*-methyl derivative might interact with ergosterol biosynthesis in fungi or cholesterol biosynthesis in human cells by mimicking one of the numerous carbocationic HEIs [10, 13] of the enzymatic steps in the post-squalene part of sterol biosynthesis.

## Results and Discussion

Grundmann’s ketone (**1**) [14] was obtained in a high yield by ozonolysis of cholecalciferol (vitamin D_3_), by instead using our improved workup procedure (treatment of the ozonolysis mixture with water, followed by extraction with pentane [10]). For the annulation of the pyridine ring we investigated two different approaches.

In the style of Gladiali’s pyridoannulation protocol [15], ketone **1** was first converted to *N*,*N*-dimethylhydrazone **2** by condensation with *N*,*N*-dimethylhydrazine in ethanol, and significant conversion was only obtained upon microwave irradiation. In the next step, **2** was converted to its kinetic lithium enolate with lithium diisopropylamide (LDA), followed by trapping with 2-(2-bromoethyl)-1,3-dioxolane to give regioselectively the double-masked 1,5-dicarbonyl compound **3**. The absolute configuration at the newly generated stereo-center at C-5 could not be determined by NMR, but was of minor interest, since it was to be destroyed in the next step anyway. Finally, refluxing intermediate **3** in glacial acetic acid under microwave irradiation produced the annulated pyridine **4** in a 29% yield. The mechanism of this cyclization comprises *in situ* opening of the acetal and extrusion of dimethylamine [15]. This procedure produced the target pyridine **4** in a 5.3% overall yield in three steps from Grundmann’s ketone (**1**). The structure of **4** was confirmed by the typical ^1^H- and ^13^C-NMR resonances of a 2,3-disubstituted pyridine ring and fitting MS data.

Due to the poor overall yield of the approach described above, we also explored the pyridoannulation protocol of Koyama [16] involving the thermolysis of an oxime *O*-allyl ether. Grundmann’s ketone (**1**) was easily converted into the oxime ether **5** by condensation with *O*-allylhydroxylamine. The thermolysis of **5** to yield the target pyridine **4** turned out to be the critical step in this approach. Heating neat **5** in a sealed glass tube at 180 °C, as described in [16], did not result in the target product, and the same disappointing result was obtained upon refluxing the oxime ether in naphthalene (218 °C) or heating at 220 °C in benzene in a sealed tube for a prolonged amount of time (20 h). Finally, we found that microwave irradiation of neat **5** under air and pressure in a sealed tube led to the conversion to the annulated pyridine **4**. Careful optimization of the reaction conditions led to the conclusion that high temperature (220 °C) combined with a short reaction time (5 min) gives the best yield (29%). With this protocol, pyridine **4** was obtained in a 23% overall yield in two steps from Grundmann’s ketone (**1**).

Due to its moderate basicity, pyridine **4** is not likely to be protonated to a significant extent under physiological conditions (pH 7.4). But a positive charge at the nitrogen would be a prerequisite for mimicking cationic HEIs in sterol biosynthesis. In order to introduce a positive charge into the molecule, we converted **4** into the *N*-methylpyridinium iodide **6** by reaction with methyl iodide.

The resulting azasteroid derivatives **4** and **6** were tested in an agar diffusion assay against a panel of bacteria and fungi (Tab. 1), and in an MTT assay [17] for cytotoxicity on human cells.

In the test for antimicrobial activity, compared to the antibiotic tetracycline and the antifungal clotrimazole, pyridine derivative **4** was found to be completely inactive. In contrast, the *N*-methylpyridinium salt **6** showed strong activity against Gram-positive bacteria, yeasts, and the dermatophyte *Hyphopichia burtonii*. This pattern of selectivity parallels the pattern of the antiseptic cetylpyridinium chloride [18], which is known to be highly active against Gram-positive bacteria and yeasts, but has a gap in its effectiveness against Gram-negative pathogens. Notably, **6** showed significantly higher antimicrobial activity than cetylpyridinium chloride.

The cytotoxicity of the compounds was determined in a MTT assay on HL-60 cells (human leukemia cell line) using the method of Mosmann [17]. The reference drug cisplatin gave an IC_50_ = 5 μM in this test. Pyridine **4** showed only very weak cytotoxicity (IC_50_ = 16 μM), whereas the pyridinium salt **6** was found to exhibit very strong cytotoxicity (IC_50_ = 0.8 μM).

Finally, both compounds were subjected to the assays for the detection of enzyme inhibition in the post-squalene part of cholesterol biosynthesis (on the human cell line HL-60 [19]) and ergosterol biosynthesis (model strain: *Yarrowia lipolytica*[20]), which have been worked out by our group previously. Both **4** and **6** were devoid of effects on sterol biosynthesis in both assays. Analysis of the sterol patterns after appropriate times of incubation showed that the sterol patterns of the cell lines were unchanged.

## Conclusion

In conclusion, we worked out a convenient approach to 7-aza-des-*A*-steroids, which were intended to inhibit enzymes in ergosterol or cholesterol biosynthesis. Surprisingly, pyridine **4** as well as its *N*-methylpyridinium analogue **6** did not interfere at all with sterol biosynthesis. In a test for antimicrobial activities, the pyridine derivative **4** was inactive. In contrast, the pyridinium salt **6** showed an activity pattern quite similar to the one of cetylpyridinium chloride, but its activity was significantly higher compared to this reference drug. This allows the conclusion that the antimicrobial activity, and probably also the significant cytotoxic activity against a human cancer cell line, of **6** are most likely due to its detergent-like structure. The sterol-like partial structure of **6** might support the incorporation of this compound into cell membranes and concomitant disturbance of membrane integrity, thus leading to the observed effects.

## Experimental

### General

Mass spectra: Hewlett Packard 5989 A, EI at 70 eV, chemical ionisation (CI) with CH_4_ (300 eV); NMR: Jeol GSX 400 (^1^H: 400 MHz, ^13^C: 100 MHz); melting points: Büchi Melting Point B-540 (not corrected); flash column chromatography (FCC): silica gel 60 (230–400 mesh, E. Merck, Darmstadt, Germany); microwave-assisted reactions were performed in a CEM Discovery reactor (CEM, Matthews, USA).

### 2-[(1R,3aR,7aR)-1-[(1R)-(1,5-Dimethylhexyl]-7a-methyloctahydro-4H-inden-4-ylidene]-1,1-dimethylhydrazine (2)

A solution of 3.00 g (11.3 mmol) Grundmann’s ketone (**1**) and 3.44 mL (45.4 mmol) of *N*,*N*-dimethylhydrazine in 30 mL EtOH was heated under microwave irradiation (150 W) for 4 h, then treated with 50 mL of hydrochloric acid (10%), and extracted with CH_2_Cl_2_ (3 × 50 mL). The combined organic layers were dried over MgSO_4_ and the solvent was evaporated. The residue was purified by FCC (isohexane/ethyl acetate 4:1) to give 1.62 g (47%) of **2** as a pale yellow oil. ^1^H-NMR (CDCl_3_): δ (ppm) = 2.36 (s, 3H, N-CH_3_, 2.35 (s, 3H, N-CH_3_), 2.32–2.20 (m, 1H, 3a-H), 2.15–0.97 (m, 19H, 1-H, 2-H, 3-H, 5-H, 6-H, 7-H, 1’-H, 2’-H, 3’-H, 4’-H, 5’-H), 0.96 (s, 3H, 7a-CH_3_), 0.89–0.84 (m, 9H, 1’-CH_3_, 5’-CH_3_, 6’-H). ^13^C-NMR (CDCl_3_): δ (ppm) = 170.57 (C-4), 54.96 (C-3a), 53.40 (C-1), 46.33 (C-7a), 44.93 (C-5), 39.84 (N(CH_3_)_2_), 38.39 (C-6), 36.39 (C-7), 33.38 (C-3), 32.83 (C-1’), 27.01 (C-5’), 26.39 (C-2), 23.79 (C-2’), 22.83 (7a-CH_3_), 21.78 (5’-CH_3_), 21.49 (C-6’), 21.06 (C-3’), 19.94 (C-4’), 18.71 (1’-CH_3_). CI-MS m/z (rel. int.): 307 (100, M^+^+1), 278 (18). HR-MS (EI): calcd.: 306.3035, found: 306.3002.

### 2-{(1R,3aR,7aR)-1-[(1R)-1,5-Dimethylhexyl]-5-[2-(1,3-dioxolan-2-yl)ethyl]-7a-methyloctahydro-4H-inden-4-ylidene}-1,1-dimethylhydrazine (3)

3.00 mL (5.28 mmol) of LDA solution (1.76 M in THF) was added to 20 mL of anhydrous THF under N_2_, and the mixture was cooled to 0 °C. A solution of 1.62 g (5.28 mmol) of dimethylhydrazone **2** in 30 mL anhydrous THF was added dropwise with stirring. After 2 h at 0 °C, the mixture was cooled to −78 °C, and 0.62 mL (5.28 mmol) 2-(2-bromoethyl)-1,3-dioxolane were added slowly. After stirring at −78 °C for 1 h, the mixture was allowed to warm up to room temp. and quenched with 40 mL of water. The mixture was extracted with diethyl ether (3 × 50 mL). The combined organic layers were dried over MgSO_4_ and the solvent was evaporated. The residue was purified by FCC (isohexane/ethyl acetate 7:3) to give 848 mg (39%) of **3** as a pale yellow oil. ^1^H-NMR (CDCl_3_): δ (ppm) = 4.83 (t, 1H, *J* = 4.4 Hz, 3”-H), 3.99–3.91 (m, 2H, acetal-CH_2_), 3.88–3.79 (m, 2H, acetal-CH_2_), 2.50–2.43 (m, 6H, N(CH_3_)_2_), 2.41 (dd, 1H, *J* = 8.5 Hz, 5.3 Hz, 3a-H), 2.31–2.19 (m, 1H, 2”-H), 2.14–2.02 (m, 1H, 2”-H), 1.95–1.83 (m, 2H, 1”-H), 1.81–1.04 (m, 17H, 1-H, 2-H, 3-H, 5-H, 6-H, 7-H, 2’-H, 3’-H, 4’-H, 5’-H), 1.02 (s, 3H, 7a-CH_3_), 0.93 (m, 1H, 1’-H), 0.88–0.83 (m, 9H, 1’-CH_3_, 5’-CH_3_, 6’-H). ^13^C-NMR (CDCl_3_): δ (ppm) = 174.90 (C-4), 104.23 (C-3”), 64.87 (acetal-CH_2_), 64.84 (acetal-CH_2_), 58.79 (C-3a), 54.43 (C-1), 49.15 (C-7a), 48.42 (C-5), 39.84 (N(CH_3_)_2_), 39.38 (C-6), 36.57 (C-7), 35.44 (C-3), 34.17 (C-1’), 31.38 (C-5’), 28.27 (C-2), 27.96 (C-2’), 27.54 (C-2”), 26.01 (C-1”), 24.17 (7a-CH_3_), 22.78 (5’-CH_3_), 22.51 (C-6’), 22.01 (C-3’), 21.57 (C-4’), 19.02 (1’-CH_3_). CI-MS m/z (rel. int.): 407 (100, M^+^+1). HR-MS (EI): calcd.: 406.3559, found: 406.3185.

### (6aR,7R,9aR)-7-[(1R)-1,5-Dimethylhexyl]-6a-methyl-6,6a,7,8,9,9a-hexahydro-5H-cyclopenta[h]quinoline (7-Aza-19-nor-des-A-cholesta-5,7,9-triene, 4)

#### Method 1

A solution of 848 mg (2.08 mmol) of **3** in 5 mL glacial acetic acid was refluxed under microwave irradiation (300 W) for 3 h, then neutralized carefully with saturated Na_2_CO_3_ solution, and extracted with CH_2_Cl_2_ (3 × 50 mL). The combined organic layers were dried over MgSO_4_ and the solvent was evaporated. The residue was purified by FCC (isohexane/ethyl acetate 9:1) to give 182 mg (29%) of **4** as a light brown oil.

#### Method 2

860 mg (2.69 mmol) of the *O*-allyl oxime **5** was heated in a closed vessel without solvent under microwave irradiation (300 W, T_max_ = 290 °C) for 5 min. The product was purified by FCC (isohexane/ethyl acetate 9:1) to give 230 mg (29%) of **4** as a light brown oil.

^1^H-NMR (CDCl_3_): δ (ppm) = 8.36 (d, 1H, *J* = 4.8 Hz, 2-H), 7.31 (d, 1H, *J* = 7.6 Hz, 4-H), 6.95 (dd, 1H, *J* = 7.6 Hz, 4.7 Hz, 3-H), 2.79 (t, 1H, *J* = 8.5 Hz, 9a-H), 2.67–2.65 (m, 2H, 5-H), 2.29–2.22 (m, 1H, 6-H_a_), 1.79–1.73 (m, 2H, 8-H_a_, 9-H_a_), 1.49–1.32 (m, 7H, 1’-H, 7-H, 9-H_b_, 8-H_b_, 5’-H, 2’-H), 1.16–1.01 (m, 8H, 3’-H, 4’-H, 6-H_b_, 6a-CH_3_), 0.94 (d, 3H, *J* = 6.5 Hz, 1’-CH_3_), 0.84 (d, 3H, *J* = 6.6 Hz, 6’-H), 0.82 (d, 3H, *J* = 6.6 Hz, 5’-CH_3_). ^13^C-NMR (CDCl_3_): δ (ppm) = 161.44 (C-9b), 147.26 (C-2), 135.48 (C-4), 131.67 (C-4a), 120.49 (C-3), 54.90 (C-9a), 53.27 (C-7), 42.52 (C-6a), 39.56 (C-4’), 35.71 (C-2’), 35.35 (C-8), 33.83 (C-1’), 31.73 (C-6) 28.88 (C-9), 28.06 (C-5’), 25.97 (C-5), 24.44 (C-3’), 23.40 (6a-CH_3_), 22.89 (C-6’), 22.64 (5’-CH_3_) 19.72 (1’-CH_3_). CI-MS m/z (rel. int.): 300 (100, M^+^+1), 159 (12). HR-MS (EI): calcd.: 299.2613, found: 299.2608.

### (1R,3aR,7aR)-1-[(1R)-1,5-Dimethylhexyl]-7a-methyloctahydro-4H-inden-4-one O-allyloxime (5)

3.03 g (11.5 mmol) of Grundmann’s ketone (**1**), 2.15 g (19.6 mmol) of *O*-allylhydroxylamine hydrochloride, 1.34 g (22.6 mmol) of sodium acetate, and 2.40 g (22.6 mmol) of Na_2_CO_3_ were dispersed in 25 mL ethanol. This mixture was refluxed for 6 h, stirred for another 12 h at ambient temp., and then evaporated to dryness. The residue was taken up in 60 mL CH_2_Cl_2_ and washed with 3 × 50 mL hydrochloric acid (1.5%). The organic layer was dried over MgSO_4_ and the solvent was evaporated. The residue was purified by FCC (isohexane/ethyl acetate 47:3) to give 2.93 g (80%) of **5** as a colourless oil. ^1^H-NMR (CDCl_3_): δ (ppm) = 6.02–5.95 (m, 1H, 2”-H), 5.29–5.15 (m, 2H, 3”-H), 4.52 (m, 2H, 1”-H), 2.30–2.25 (m, 1H, 3a-H), 2.06–1.99 (m, 1H, 1-H), 1.88–1.82 (m, 1H, 3-H), 1.62–1.46 (m, 9 H, 6-H, 5-H, 3-H, 3’-H, 2’-H), 1.33–1.24 (m, 5H, 2-H, 4’-H, 1’-H), 1.12–1.09 (m, 3H, 7-H, 5’-H), 0.96 (s, 3H, 7a-CH_3_), 0.89–0.85 (m, 9 H, 1’-CH_3_, 5’-CH_3_, 6’-H). ^13^C-NMR (CDCl_3_): δ (ppm) = 160.63 (C-4), 134.72 (C-2”), 116.91 (C-3”), 74.19 (C-1”), 56.08 (C-3a), 52.68 (C-1), 46.59 (C-7a), 39.54 (C-5), 37.64 (C-6), 36.17 (C-7), 35.16 (C-3), 34.04 (C-1’), 28.03 (C-5’), 24.85 (C-2), 23.90 (C-2’), 23.13 (7a-CH_3_), 23.08 (5’-CH_3_), 22.65 (C-6’), 22.08 (C-3’), 19.65 (C-4’), 18.90 (1’-CH_3_). CI-MS m/z (rel. int.): 320 (100, M^+^+1), 262 (50). HR-MS (EI): calcd.: 319.2875, found: 319.2867.

### (6aR,7R,9aR)-7-[(1R)-1,5-Dimethylhexyl]-1,6a-dimethyl-6,6a,7,8,9,9a-hexahydro-5H-cyclopenta[h]quinolinium iodide (6)

126 mg (0.42 mmol) of pyridine **5** were dissolved in a minimal quantity of acetone and treated with 0.65 ml (1.04 mmol) of methyl iodide (caution! highly toxic agent!). The mixture was stirred in a well-ventilated hood for 16 h at ambient temp., then the precipitate was collected by filtration and recrystallized from ethanol to give 99 mg (53%) of **6** as white crystals. M.p. 218 °C. ^1^H-NMR (CDCl_3_): δ (ppm) = 9.43 (d, 1H, *J* = 5.9 Hz, 2-H), 8.17 (d, 1H, *J* = 7.8 Hz, 4-H), 7.83 (t, 1H, *J* = 7.0 Hz, 3-H), 4.56 (s, 3H, 1-CH_3_), 3.21 (t, 1H, *J* = 9.4 Hz, 9a-H), 2.89 (m, 2H, 5-H), 2.48–2.38 (m, 1H, 6-H_a_), 2.08 (m, 1H, 8-H_a_), 2.00–1.90 (m, 1H, 9-H_a_), 1.67–1.35 (m, 7H, 1’-H, 7-H, 9-H_b_, 8-H_b_, 5’-H, 2’-H), 1.23–1.10 (m, 8H, 3’-H, 4’-H, 6-H_b_, 6a-CH_3_), 0.99 (d, 3H, *J* = 6.0 Hz, 1’-CH_3_), 0.86 (d, 6H, *J* = 6.5 Hz, 5’-CH_3_, 6’-H). ^13^C-NMR (CDCl_3_): δ (ppm) = 157.70 (C-9b), 144.96 (C-2), 143.61 (C-4), 138.13 (C-4a), 123.65 (C-3), 51.10 (C-7), 48.58 (C-9a), 45.76 (1-CH_3_), 43.42 (C-6a), 38.35 (C-4’), 34.39 (C-2’), 33.34 (C-8), 31.91 (C-1’), 30.83 (C-6), 27.00 (C-5’), 26.87 (C-9), 25.07 (C-5), 24.16 (6a-CH_3_), 23.32 (C-3’), 21.77 (C-6’), 21.53 (5’-CH_3_), 18.81 (1’-CH_3_). CI-MS m/z (rel. int.): 314 (M^+^, cation, 54), 300 (100). HR-MS (EI): calcd.: 313.2769, found: 313.2726.

### Agar diffusion assay

The assay was performed as described in detail previously [21] using the microorganisms listed in Table 1. Paper discs (6 mm diameter) were impregnated with 30 μg of each test substance or the reference drugs. The diameters of the zones of inhibition were measured manually. The experiments were performed in triplicate. The antibiotic tetracycline-HCl and the antifungal clotrimazole were used as reference drugs.

### MTT assay

The assay originally worked out by Mosmann [17] was performed as described in detail previously [21] using HL-60 cells. The experiments were performed in triplicate. Cisplatin was used as the reference drug.

### Screening for inhibition of cholesterol biosynthesis

The qualitative assay was performed as described in detail previously [19] using HL-60 cells.

### Screening for inhibition of ergosterol biosynthesis

The qualitative assay was performed as described in detail recently [20] using the yeast *Yarrowia lipolytica* as the test strain.

## Figures and Tables

**Sch. 1.**
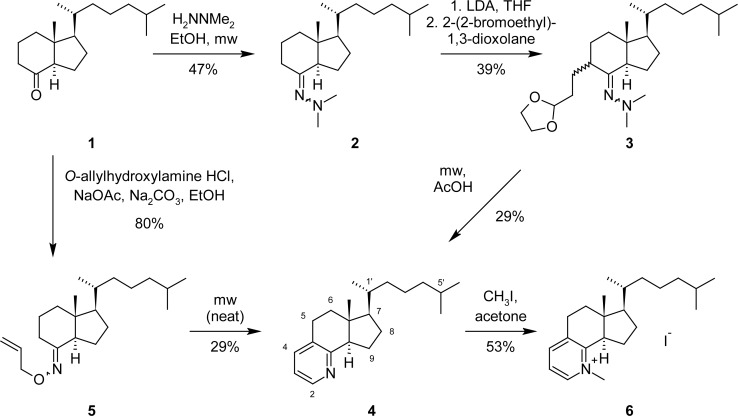
Two approaches to the target compounds **4** and **6.**

**Tab. 1.** Antimicrobial activities determined in an agar diffusion assay^a^

**Compound**	**Bacteria**^b^				**Fungi**^c^			

	***E. coli***	***Pseud. aer.***	***Staph. euq.***	***Strept. ent.***	***Yarr. lipol.***	***Cand. glabr.***	***Hyph. burt.***	***Asp. niger***
**4**	–^d^	–	–	–	–	–	–	–
**6**	7	–	30	22	32	30	20	7
Tetracycline-HCl	30	25	42	25	n.d.^e^	n.d.	n.d.	n.d.
Clotrimazole	n.d.	n.d.	n.d.	n.d.	28	15	25	30
Cetylpyridinium chloride	–	–	–	10	8	9	–	7

a Diameters of inhibition zones in mm, mean of 3 runs each.

b Gram-negative bacteria: E. coli = *Escherichia coli*, Pseud. aer. = *Pseudomonas aeruginosa*; Gram-positive bacteria: *Staph. euq.* = *Staphylococcus equorum*, *Strept. ent.* = *Streptococcus entericus*.

c Yeasts: *Yarr. lipol.* = *Yarrowia lipolytica*, *Cand. glabr.* = *Candida glabrata*; dermatophyte: *Hyph. burt.* = *Hyphopichia burtonii*; mould: *Asp. niger* = *Aspergillus niger*.

d No zone of inhibition is detectable.

e Not determined.
